# Comparison of Duration of Postapproval vs Pivotal Trials for Therapeutic Agents Granted US Food and Drug Administration Accelerated Approval, 2009-2018

**DOI:** 10.1001/jamanetworkopen.2021.33601

**Published:** 2021-11-09

**Authors:** Joshua D. Wallach, Reshma Ramachandran, Till Bruckner, Joseph S. Ross

**Affiliations:** 1 Department of Environmental Health Sciences, Yale School of Public Health, New Haven, Connecticut; 2 National Clinician Scholars Program, Department of Internal Medicine, Yale School of Medicine, New Haven, Connecticut; 3 Department of Internal Medicine, Veterans Affairs Connecticut Healthcare System and Yale University, West Haven, Connecticut; 4 QUEST Center, Berlin Institute of Health, Charité, Universitätsmedizin Berlin, Berlin, Germany; 5 TranspariMED, Bristol, United Kingdom; 6 Section of General Internal Medicine, Department of Internal Medicine, Yale School of Medicine, New Haven, Connecticut

## Abstract

This cross-sectional study compares the duration of postapproval trials with that of the pivotal trials used as the basis for the US Food and Drug Administration’s (FDA’s) approval for all indications receiving accelerated approval from 2009-2018.

## Introduction

Under the US Food and Drug Administration’s (FDA’s) accelerated approval program, therapeutic agents that address serious or life-threatening disease can receive conditional approval on the basis of trials that use surrogate markers that are reasonably likely to predict clinical benefit.^1^ Although drug sponsors are required to conduct postapproval trials to confirm clinical benefit, the amount of time sponsors are granted by the FDA to complete these studies has come under increased scrutiny after the recent accelerated approval of aducanumab for Alzheimer disease,^2^ which included a 9-year deadline for the completion of the confirmatory trial. Better understanding whether postapproval trial durations, which are generally expected to measure clinical outcomes and be longer than pivotal trials focused on surrogate markers, justify the study timelines set by the FDA is critical because these drugs can remain on the market for an extended period without confirmatory evidence. Therefore, we compared the duration of postapproval trials with that of the pivotal trials used as the basis for the FDA’s approval, as well as FDA-established postapproval trial results reporting deadlines, for all indications receiving accelerated approval from January 1, 2009, through December 31, 2018.

## Methods

In this cross-sectional study, we used the Drugs@FDA database to identify all new drugs and biologics granted accelerated approval by the FDA from January 1, 2009, through December 31, 2018. This study did not require institutional review board approval because it was based on publicly available information, in accordance with 45 CFR §46. Informed consent was not needed because no patient data were used. This study followed the Strengthening the Reporting of Observational Studies in Epidemiology (STROBE) reporting guideline. For each therapeutic agent, we used previously described approaches to identify pivotal and postapproval trials designed to confirm efficacy,^3,4,5^ classify postapproval trials as either new or ongoing at the time of approval,^4^ identify FDA-established postapproval trial results reporting deadlines,^4,6^ and locate ClinicalTrials.gov registrations and publications.^4^ We used FDA labels/letters and medical reviews, ClinicalTrials.gov registrations, and publications to identify pivotal and postapproval trial primary outcomes and the timing of their ascertainment (ie, median duration of follow-up or response for event-driven trials). For indications with more than 1 pivotal study and/or postapproval trial, we calculated the median of the trial durations. For each indication, we then calculated the difference between the durations of the postapproval and pivotal trials. As suggested during the peer review of this manuscript, we also estimated trial durations by taking the difference between the actual or estimated study start and primary completion dates available for all trials registered on ClinicalTrials.gov. These data analyses were conducted from July 1 to September 1, 2021, using Excel (Microsoft, Inc).

## Results

From Jauary 1, 2009, through December 31, 2018, the FDA approved 41 new therapeutic agents for 45 indications via the accelerated approval program. Of these, information on trial duration could be ascertained for 31 therapeutic agents (76%) approved for 32 indications (76%). Of 32 indications approved, 21 (66%) were for drugs, 11 (34%) were for biologics, and 27 (84%) were for therapeutic agents related to cancer and hematology (Table 1).

**Table 1.** Characteristics of 32 Indications Receiving US Food and Drug Administration Accelerated Approval, 2009-2018^a^

Indication characteristic	No. (%)
Class	
Drug	21 (66)
Biologic	11 (34)
Therapeutic area	
Cancer and hematology	27 (84)
Other	5 (16)
Priority review	
Yes	28 (88)
No	4 (12)
Fast track	
Yes	17 (53)
No	15 (47)
Breakthrough therapy	
Yes	17 (53)
No	15 (47)
Orphan drug designation	
Yes	29 (91)
No	3 (9)
Converted from accelerated to regular approval	
Yes	19 (59)
No	13 (41)
Total sample size per indication, median (IQR)	
Pivotal trial	164 (105-235)
Postapproval confirmatory trial	520 (386-709)

^a^ For 1 therapeutic agent (Iclusig [ponatinib hydrochloride]; Takeda Pharmaceutical Company Limited), 1 pivotal and 1 postapproval confirmatory trial evaluated 2 indications (chronic myeloid leukemia and acute lymphoblastic leukemia). Therefore, we counted this as 1 indication approval.

Overall, the median pivotal trial duration was 10 months (range, 0-42 months) and the postapproval trial duration was 17 months (0-72 months) (median difference, 5 months [range, −6 to 36 months]) (Table 2). The median time from approval to FDA-established postapproval trial results reporting deadlines for sponsors was 50 months (3-116 months), a median of 30 months (range, −19 to 106 months) more than the postapproval trial durations.

**Table 2.** Pivotal and Postapproval Trial Durations

Trial type	Trial duration, median (range), mo										
	Primary analyses^a^					Sensitivity analyses^b^				
	Postapproval trial status at approval		Postapproval end point type		Overall	Postapproval trial status at approval		Postapproval end point type		Overall
	New (n = 17)	Ongoing (n = 15)	Surrogate (n = 19)	Clinical (n = 13)		New (n = 20)	Ongoing (n = 16)	Surrogate (n = 22)	Clinical (n = 14)	
Pivotal	9 (0 to 42)	10 (2 to 29)	10 (2 to 26)	8 (0 to 42)	10 (0 to 42)	32 (12 to 92)	55 (10 to 117)	39 (10 to 117)	29 (12 to 92)	37 (10 to 117)
Postapproval	19 (4 to 72)	15 (0 to 41)	17 (0 to 36)	17 (4 to 72)	17 (0 to 72)	43 (4 to 111)	45 (19 to 120)	50 (14 to 11)	37 (4 to 120)	44 (4 to 120)
Difference^c^	7 (−2 to 30)	4 (−6 to 36)	5 (−3 to 24)	6 (−6 to 36)	5 (−6 to 36)	2 (−57 to 77)	1 (−85 to 97)	1.5 (−85 to 77)	−5.5 (−57 to 97)	1 (−85 to 97)

^a^ Duration based on timing of primary end point ascertainment among 32 indications. This included 5 indications with ongoing trials for which estimated trial durations were used.

^b^ Duration based on ClinicalTrials.gov study start and primary completion dates among 37 indications. This included 7 indications with ongoing trials for which only estimated primary completion dates were available.

^c^ Difference was calculated by subtracting the pivotal trial duration from the postapproval trial duration.

Median pivotal and postapproval trial durations were consistent across indications for which the FDA required new postapproval trials vs results reporting or extended follow-up of ongoing postapproval trials and for indications for which postapproval trials used surrogate markers vs clinical outcomes as primary end points (Table 2). Among 37 indications with information on study start and completion dates on ClinicalTrials.gov, the median difference between pivotal and postapproval trial durations based on these data was 1 month (range, −85 to 97 months).

## Discussion

The findings of this cross-sectional study suggest that, for therapeutic agents receiving accelerated approval by the FDA from January 1, 2009, through December 31, 2018, median postapproval trial durations were not much longer than pivotal trial durations. These findings raise questions about the use of the accelerated approval program for certain therapeutic agents, especially if postapproval confirmatory trials neither consistently evaluate clinical outcomes nor are much longer than pivotal trials using surrogate end points.

Limitations of this study include the reliance on publicly available data, the inability to account for postapproval trial recruitment challenges or other reporting deadline changes, and that these results may not be generalizable across all therapeutic areas. Nevertheless, this study’s findings suggest that prolonged results reporting deadlines may not be justified given the marginal differences in pivotal and postapproval trial durations of therapeutic indications recently receiving accelerated approval by the FDA.
